# Body mass index distinctly modulates the associations between *Alistipes* and CRP/IL-6 in metabolic and lupus inflammatory features

**DOI:** 10.1371/journal.pone.0335452

**Published:** 2025-11-25

**Authors:** Lourdes Chero-Sandoval, Andrea Higuera-Gómez, Begoña de Cuevillas, Raquel Castejón, María Martínez-Urbistondo, Susana Mellor-Pita, Víctor Moreno-Torres, Daniel de Luis, Amanda Cuevas-Sierra, J. Alfredo Martínez

**Affiliations:** 1 Precision Nutrition and Cardiometabolic Health, IMDEA-Food Institute (Madrid Institute for Advanced Studies), Campus of International Excellence (CEI) UAM+CSIC, Madrid, Spain; 2 Department of Endocrinology and Nutrition, University Clinical Hospital, University of Valladolid, Valladolid, Spain; 3 Internal Medicine Service, Puerta de Hierro Majadahonda University Hospital, Madrid, Spain; 4 Centre of Endocrinology and Nutrition, University of Valladolid, Valladolid, Spain; 5 CIBERobn Physiopathology of Obesity and Nutrition, Institute of Health Carlos III (ISCIII), Madrid, Spain; University of Michigan, UNITED STATES OF AMERICA

## Abstract

Several investigations have documented relationships between body mass index (BMI) and gut microbiota composition in the context of inflammation. However, the precise interaction between BMI and gut microbiota influencing inflammatory markers is still unclear, presenting a challenge for personalized interventions. This study aimed to analyze anthropometric, biochemical and inflammatory variables of participants with systemic lupus erythematosus (SLE) compared to those with low-grade metabolic inflammation (MI), as well as to elucidate the impact of gut microbiota composition, particularly *Alistipes*, in relation to adiposity, as assessed by BMI, on inflammatory markers within the METAINFLAMATION cohort. A total of 127 adults diagnosed with both diseases, categorized according to the WHO definition of obesity (BMI ≥ 30 kg/m^2^) and phenotypically analyzed by 16S sequencing of fecal samples. The results showed that patients with low-grade MI had higher anthropometric values, glycosylated hemoglobin and triglyceride levels. On the other hand, patients with SLE and high BMI showed elevated insulin, CRP, and fibrinogen levels, alongside significant changes in *Alistipes* abundance. Notably, CRP levels were influenced by both BMI and Alistipes, particularly in lupus individuals with higher BMI, and elevated IL-6 was associated with higher CRP in this subgroup. CRP levels are influenced by BMI and *Alistipes shahii* abundance, especially in lupus individuals with higher BMI. In addition, elevated IL-6 concentrations are significantly associated with more elevated CRP levels in SLE individuals with higher BMI. These findings showed that CRP levels are influenced by BMI and Alistipes, particularly in lupus individuals with higher BMI and highlight the need for personalized approaches based on body composition, inflammatory markers and gut microbiota in patients with lupus and obesity for improving clinical management and outcomes.

## Introduction

Inflammation is a fundamental biological response to harmful stimuli, typically self-limiting and resolved upon elimination of the trigger [1]. When persistent or dysregulated, however, it evolves into a chronic state [2]. This chronic inflammation is characterized by an impaired and sustained immune response, involving complex biological processes driven by immunological and homeostatic mechanisms [3].

Chronic inflammation may arise in a variety of pathological contexts—including autoimmune diseases, obesity, type 2 diabetes, cancer, chronic infections, and neurodegenerative disorders—each with distinct immunological and metabolic features [4,5]. While sustained inflammation is a hallmark across these conditions, the underlying mechanisms and systemic consequences vary substantially [1].

Autoimmune-driven inflammation, for example, is primarily fueled by aberrant immune recognition and loss of self-tolerance. Systemic lupus erythematosus (SLE) is a prototypical autoimmune disease characterized by widespread inflammation and multi-organ involvement [6]. In SLE, the immune system generates autoantibodies against nuclear and cytoplasmic antigens, leading to immune complex deposition, complement activation, and persistent inflammatory responses that drive disease progression [7].

On the other hand, excess of body weight contributes to chronic inflammation through distinct but interconnected mechanisms [8]. Adipose tissue expansion promotes local hypoxia and cellular stress, which in turn recruit immune cells—particularly pro-inflammatory macrophages—and stimulate the production of cytokines such as tumor necrosis factor-alpha (TNF-α) and interleukin-6 (IL-6), while decreasing levels of anti-inflammatory adipokines like adiponectin [9]. In this context, low-grade metabolic inflammation (MI)—commonly associated with overweight, obesity, and insulin resistance—is driven by adipose tissue dysfunction and the chronic release of pro-inflammatory mediators, leading to a persistent, mild systemic inflammatory state [10].

Beyond disease-specific mechanisms, gut microbiota has emerged as a crucial modulator of chronic inflammation [11]. The intestinal microbiota plays an active role in immune system regulation, metabolic homeostasis, and epithelial barrier integrity [12]. Dysbiosis—defined as an imbalance in microbial composition and function—can disrupt gut barrier function, facilitating the translocation of microbial-derived molecules such as lipopolysaccharide (LPS), which activate innate immune receptors and perpetuate systemic inflammation [13]. This microbial-driven inflammatory signaling can exacerbate the chronic immune activation already present in conditions like SLE and MI. Moreover, specific microbial taxa have been linked to either pro-inflammatory or anti-inflammatory effects, suggesting that the composition of the gut microbiota may influence both the intensity and the clinical consequences of chronic inflammation [14–17].

In summary, the interplay between adiposity, inflammation and gut microbiota underscores the need for integrative therapeutic strategies that address both body weight control and gut microbiota composition, as these approaches could be key to mitigating chronic inflammation in lupus and obesity patients [18]. In the era of omics data, where high-throughput technologies provide unprecedented insight into genomic, transcriptomic, proteomic, metabolomic, and microbiome profiles, chronic inflammatory diseases—whether autoimmune or metabolic in origin—can now be studied with greater resolution and contextual depth [19,20]. This wealth of patient-derived molecular information opens the door to more personalized and mechanism-based interventions, allowing clinicians and researchers to identify distinct inflammatory signatures, stratify patients according to their immunometabolic profiles, and ultimately tailor therapies to individual disease mechanisms [21]. By incorporating traditional variables such as body weight and novel biomarkers such as the gut microbiota into clinical frameworks, we can enhance both treatment precision and long-term disease management in complex inflammatory conditions such as SLE and obesity [22].

Therefore, the aim of this study was to evaluate the impact of BMI, as assessed by BMI, on anthropometric, biochemical, and inflammatory variables in patients with MI and SLE, and to investigate the modulatory role of gut microbiota on inflammatory markers in these two inflammatory conditions.

## Materials and methods

### Study design and participants

This study is part of the “METAINFLAMMATION-CM” project (reference Y2020/BIO-6600), which aims to investigate chronic inflammation across various medical conditions and to evaluate the influence of excess body weight on inflammatory processes. The study was designed as a prospective, controlled investigation and was conducted in accordance with the principles of the Declaration of Helsinki. Ethical approval was granted by the Research Ethics Committee of the Hospital Universitario Puerta de Hierro Majadahonda (protocol number PI 164−21).

### Participants

Participants were recruited between January 2022 and June 2023 at the Internal Medicine Service of Hospital Universitario Puerta de Hierro Majadahonda in Madrid, Spain. All individuals provided written informed consent prior to inclusion. Data collection adhered strictly to approved ethical guidelines and protocols, including anthropometric measurements, body composition analysis, biochemical assays, and relevant clinical evaluations.

This study analyzed a subsample of 127 adults from the METAINFLAMMATION-CM cohort, including both male and female participants of Caucasian and Hispanic origin (see flowchart in Supplementary S1 Fig). Participants were categorized into two mutually exclusive groups based on clinical diagnosis: low-grade metabolic inflammation (MI) and systemic lupus erythematosus (SLE), both representing complex inflammatory conditions. The MI group consisted of individuals diagnosed with obesity and metabolic syndrome according to World Health Organization (WHO) and National Cholesterol Education Program (NCEP) criteria, respectively, as these are recognized manifestations of low-grade metabolic inflammation [23]. The SLE group was defined according to the classification criteria of the European League Against Rheumatism (EULAR) and the American College of Rheumatology (ACR) [24]. Body mass index (BMI) was calculated for all participants, with obesity defined as BMI ≥ 30 kg/m² following WHO standards [23]

### Inclusion and exclusion criteria

Participants had to be over 18 years of age, have a BMI between 17.01 kg/m² and 51.35 kg/m², and have a confirmed diagnosis of low-grade MI or SLE by the medical staff at the Hospital Universitario Puerta de Hierro Majadahonda in Madrid, Spain. Participants included in MI group presented obesity and metabolic syndrome with characteristics such as excess body fat, glucose intolerance, central obesity, dyslipidemia and hypertension according to ATP III criteria [25]. SLE patients were selected in stable condition and under supervised medical treatment, and clinical parameters such as serological activity, active disease, complete remission and maintenance of low disease activity were assessed to ensure homogeneity of diagnosis and treatment [26–28]. Only those who provided fecal samples and were classified as “low BMI” or “high BMI” according to WHO criteria were included [23]. Those with severe psychiatric disorders, use of medications that alter body weight, difficulties in scheduling appointments, pregnancy, breastfeeding, recent changes in medication, and consumption of probiotics, patients with lower Systemic Lupus Erythematosus Disease Activity Index (SLEDAI-2K) and Systemic Lupus International Collaborative Clinics (SLICC) criteria, antibiotics or drugs that affect the gut microbiota three weeks prior to stool sample collection were excluded.

### Anthropometrics and body composition

Anthropometric measurements were performed by expert dieticians using validated techniques [29]. Body weight and body composition, including muscle mass, body fat, visceral fat, bone mass and body water were assessed with a bioimpedance scale (TANITA SC-330; Tanita Corporation, Japan). Waist circumference was measured with a standard tape measure following established protocols. BMI was calculated by dividing body weight by the square of height (kg/m²) [30].

### Biochemical data

Fasting blood samples were collected by venipuncture and analyzed using a SYSMEX XN-20 automated hematology analyzer (Roche, Basel, Switzerland), following validated procedures. Leukocytes, lymphocytes, neutrophils, monocytes, hemoglobin, hematocrit, platelets and red cell distribution width (RDW) were assessed, and the neutrophil/lymphocyte ratio was calculated [29]. Routine biochemical markers such as glucose, total cholesterol, glycosylated hemoglobin, ferritin, high density lipoprotein cholesterol (HDL), low density lipoprotein cholesterol (LDL), triglycerides and aPTT (activated partial thromboplastin time, a marker of coagulation activity) were measured with a quality-controlled autoanalyzer (Atellica™ Solution) following standardized hospital protocols [37]. In addition, C-Reactive Protein (CRP), fibrinogen, insulin, D-dimer, N-terminal pro B-type Natriuretic Peptide (NT ProBNP) and IL-6 were assessed using mainly ELISA (Sigma-Aldrich ELISA Kit) according to suppliers’ indications [29,30].

### Metagenomic analysis

Fecal samples were collected with OMNIgene® -GUT kits (DNA Genotek, Ottawa, Canada) according to the supplier’s instructions. Bacterial DNA was isolated using the QIAamp® kit (Qiagen, Hilden, Germany) and regions V3-V4 of the 16S rRNA gene were amplified by paired-end sequencing on the MiSeq system (Illumina, San Diego, USA) at Novogene Sequencing-Europe Service (Cambridge, UK). PCR primers were specific, and reactions were performed with Phusion® High-Fidelity PCR Master Mix (New England Biolabs)(16S Amplicon PCR Forward Primer = 5 0 TCGTCGGCAGCGTCAGATGTGTATAAGAGACAGCCTACGGGNGGCWGCAG; 16S Amplicon PCR Reverse Primer = 5 0 GTCTCGTGGGCTCGGAGATGTGTATAAGAGACAGGACTACHVGGGTATCTAATCC). PCR reactions were carried out with 15 µL of Phusion® High – Fidelity PCR Master Mix (New England Biolabs); 0.2 µM of forward and reverse primers, and about 10 ng template DNA. Thermal cycling consisted of initial denaturation at 98°C for 1 min, followed by 30 cycles of denaturation at 98°C for 10 s, annealing at 50°C for 30 s, and elongation at 72°C for 30 s and 72°C for 5 min. PCR products were purified with magnetic beads and mixed in concentration-based ratios. Sequencing libraries were generated, quantified and verified before pooling and sequencing on Illumina platforms. For bioinformatics analysis, paired-end reads were assigned to samples according to their unique barcodes and truncated. The reads were merged with FLASH (V1.2.7, http://ccb.jhu.edu/software/FLASH/) [31], and filtered with FASTP (version 0.23. 1) to obtain high quality clean tags [32]. The tags were compared with the reference database (Silva database (16S/ 18S), https://www.arb-silva.de/; Unite Database using the search (https://github.com/torognes/vsearch/) to detect and remove chimera sequences [33,34]. The noise removal was performed with DADA2 or QIIME2 (Version QIIME2–202202) to obtain amplicon sequence variants (ASVs). Species annotation and phylogenetic relationship of each ASV were studied and visualized using QIIME2 software (Version QIIME2–202202) and the R software (Version 2, vegan package) [35,36].

### Statistical analysis

Quantitative variables were expressed as means (x) and standard deviations (SD), while qualitative variables were presented as counts (n) and percentages (%). The normality of the data was assessed using the Shapiro-Wilk test. The population was stratified by body mass index (BMI) into two groups: low (≤29.99 kg/m²) and high (≥30 kg/m²), based on the World Health Organization (WHO) criteria for obesity. Differences and interactions between disease type and BMI category were analyzed using a 2 × 2 factorial ANOVA design (2 disease groups × 2 BMI categories) for anthropometric, body composition, biochemical, and inflammatory variables. Analyses were performed using RStudio (version 4.3.0, Boston, MA, USA). This 2 × 2 analysis allowed assessment of the main effects of disease and BMI, as well as their interaction, as presented in the corresponding tables. For the analysis of microbiota, data were filtered by removing features with less than 4 counts and less than 2% minimum prevalence. The alpha diversity profile between different inflammatory disease types and BMI categories was calculated using the Shannon index, commonly used in biodiversity studies to compare species diversity between samples or communities, compared using t-tests and plotted with box plots. These analyses were performed with MicrobiomeAnalyst (https://www.microbiomeanalyst.ca/) [37]. For beta diversity, the Bray Curtis index was used, together with the PERMANOVA test, and the results were visualized using principal coordinate analysis (PCoA). In addition, linear discriminant analysis (LDA) effect size (LEfSe) was used to compare and visualize groups using taxonomic bar charts. Empirical analysis for Digital Gene Expression in R (EdgeR) was used to identify families with significant differences in abundance by disease type and BMI. Data were pre-normalized with TMM, and false discovery rate correction (FDR < 0.05 for statistical significance) was applied. Random Forest algorithm was used to assess the significance of predictor variables related to BMI and inflammatory disease type, using R 3.5.3 (https://www.R-project.org/) and the Random Forest package. The model employed 500 trees and used approximately two-thirds of the samples as a training set by random sampling with replacement, validating the selected features with the remaining samples. Thus, 70% of the samples were randomly chosen to train the classifier and the remaining 30% were used for validation. Interactions between bacteria, type of disease and BMI status were investigated with models of general linear regression models that introduced the corresponding interaction terms, normalizing microbiota data with centered log ratio method for regressions (CLR) [38]. Regression models were adjusted for age, sex and adherence to Mediterranean diet (avoiding bias with microbiota) and visualized using Stata 12 (StataCorp LLC, College Station, TX, USA; http://www.stata.com). The variance inflation factor (VIF) analysis for testing collinearity between independent variables was performed to ensure variable independence. Box plots between CPR, IL-6, BMI status and *Alistipes* were performed with RStudio (version 4.3.0, Boston, MA, USA), considering that the separation by levels was performed with the median of all variables. For the variable *Alistipes*, the data were divided into “low” and “high” levels considering the median of 3.41 (values of relative abundance transformed to CLR normalization recommended for regression models) of the study group. The value of *p* < 0.05 was considered statistically significant.

## Results

### Comparison of anthropometric, body composition, biochemical and inflammatory variables between participants with low-grade MI and SLE according to BMI stratification

The categorization of the population in the low-grade MI group according to the BMI resulted in 24 participants classified as “low BMI” and 46 as “high BMI”. In the SLE group, a total of 39 patients were categorized as “low BMI” and 18 as “high BMI”. Table 1 shows the different anthropometric and body composition profiles, as well as phenotypical and clinical markers among patients with low-grade MI and SLE, compared by BMI categories (low and high BMI) following a 2x2 factorial design concerning.

**Table 1:** Comparison of anthropometric measurements, body composition and clinical variables among participants with low-grade MI and SLE and according to BMI status of the population.

	Overall	Low-grade Metabolic Inflammation			Systemic Lupus Erythematosus			*P* value		
Variables	Overall (n = 127)	Low BMI (n = 24)	High BMI (n = 46)	*P* value	Low BMI (n = 39)	High BMI (n = 18)	*P* value	Group of disease	BMI categories	Interaction
**Age (years)**	56.2 (11.5)	59.9 (9.1)	59.2 (11.2)	0.76	50.1 (11.5)	56.7(10.8)	**0.04**	**<0.001**	**0.02**	0.08
**Gender = Woman (%)**	92 (72.4)	14 (58.3)	25 (54.3)	0.95	35 (89.7)	18 (100.0)	0.39	**<0.001**	0.18	0.37
**Body weight (Kg)**	81.8 (18.5)	74.8 (8.8)	96.5 (15.9)	**<0.001**	65.8 (9.6)	87.8 (16.9)	**<0.001**	**<0.001**	**<0.001**	0.95
**Body mass index (Kg/m**^**2**^)	30.2 (5.3)	27.7 (1.4)	34.2 (3.2)	**<0.001**	24.9 (3.1)	34.7 (3.8)	**<0.001**	**<0.001**	**<0.001**	**<0.01**
**Skeletal muscle mass (Kg)**	48.6 (10.2)	47.9 (7.4)	55.1 (11.8)	**0.02**	42.9 (5.8)	45.6 (7.4)	0.22	**<0.001**	**<0.001**	0.18
**Body fat (%)**	36.6 (7.8)	32.8 (4.3)	40.0 (6.6)	**<0.001**	30.9 (6.3)	45.0 (4.1)	**<0.001**	0.12	**<0.001**	**<0.01**
**Visceral fat (AU)**	11.6 (5.3)	10.6 (3.3)	15.9 (4.5)	**<0.001**	6.7 (3.7)	12.4 (2.1)	**<0.001**	**<0.001**	**<0.001**	0.82
**Bone mass (Kg)**	2.6 (0.5)	2.6 (0.4)	2.9 (0.6)	**0.02**	2.3 (0.3)	2.4 (0.4)	0.25	**<0.001**	**<0.001**	0.19
**Body water (%)**	46.1 (5.4)	47.6 (2.9)	44.5 (5.3)	**0.03**	49.7 (4.6)	40.5 (3.0)	**<0.001**	0.19	**<0.001**	**<0.001**
**Waist circumference (cm)**	104.3 (14.0)	100.9 (6.0)	115.3 (9.6)	**<0.001**	90.0 (9.6)	111.5 (9.2)	**<0.001**	**<0.001**	**<0.001**	**0.03**
**Fasting glucose (mg/dL)**	96.2 (14.9)	103.8 (18.6)	99.6 (13.5)	0.55	88.8 (8.2)	93.2 (17.2)	0.53	**<0.001**	0.25	0.11
**Glycated Hemoglobin (%)**	5.5 (0.5)	5.7 (0.5)	5.6 (0.6)	0.87	5.3 (0.4)	5.5 (0.4)	**0.04**	**<0.01**	**0.05**	0.14
**Insulin (μIU/mL)**	10.9 (7.8)	8.0 (2.9)	13.6 (9.0)	**0.01**	8.3 (5.7)	14.4 (9.8)	**0.002**	0.28	**<0.001**	0.87
**Total cholesterol(mg/dL)**	182.9 (34.7)	186.3 (33.1)	185.6 (34.4)	0.94	184.7 (33.6)	168.1 (38.4)	0.13	0.30	0.45	0.23
**HDL-cholesterol (mg/dl)**	54.7 (14.6)	53.8 (13.8)	50.3 (13.8)	0.33	60.8 (14.5)	54.0 (14.7)	0.08	<0.01	0.01	0.55
**LDL-cholesterol (U/L)**	105.1 (28.7)	108.6 (30.4)	109.0 (27.1)	0.96	105.0 (26.4)	91.1 (32.4)	0.12	0.11	0.61	0.19
**Triglycerides (mg/dl)**	115.8 (57.3)	119.7 (45.9)	127.3 (50.3)	0.56	100.1 (74.0)	115.6 (40.5)	**0.04**	0.06	0.11	0.72

Data presented as mean (x), standard deviation (SD), and *p* values. The significance threshold was set at p < 0.05 *. *P* value refers to the comparison of variables’ mean between patients with low-grade MI and SLE using t-test or Mann-Whitney test, according to the distribution of data. Low BMI refers to BMI ≤ 30 kg/m^2^ and high BMI refers to BMI ≥ 30 kg/m^2^. “Group of disease” column is the comparison of variables’ mean between the two inflammatory diseases (without considering BMI status). “BMI categories” column is the comparison of variables’ mean between the low and high BMI. Interaction column means the *P* value of comparison between the two disease groups and the BMI categories (ANOVA 2X2). AU, Arbitrary units; HDL, High Density Lipoprotein; LDL, Low Density Lipoprotein. *P* value lower than 0.05 in bold type.

To provide a clear overview of key metabolic and inflammatory traits across the two disease groups, we compiled a supplementary table presenting BMI, body fat, waist circumference, CRP, and IL-6 for all participants, stratified by disease (low-grade MI vs. SLE). This supplementary table allows visualization of baseline differences and overlaps between groups (see Supplementary S1 Table).

In the analysis of low-grade MI groups by BMI, no significant differences were observed in age or sex. As expected, participants with higher BMI showed consistently greater anthropometric and body composition values (Table 1), with the only exception being body water percentage. Biochemically, this group also exhibited elevated insulin levels, suggesting early metabolic alterations associated with higher BMI

For SLE participants, those with higher BMI were older and displayed greater body weight, fat mass, visceral adiposity, and waist circumference (Table 2). They also showed higher levels of glycated hemoglobin, triglycerides, and insulin, indicating that obesity in SLE is accompanied by more pronounced metabolic disturbances.

**Table 2:** Comparison of hematological and inflammatory variables between types of disease (low-grade metabolic inflammation and systemic lupus erythematosus) according to BMI status.

	Overall	Low-grade Metabolic Inflammation			Systemic Lupus Erythematosus			*P* value		
Variables	Overall (n = 127)	Low BMI (n = 24)	High BMI (n = 46)	*P* value	Low BMI (n = 39)	High BMI (n = 18)	*P* value	Group of disease	BMI categories	Interaction
**Leukocytes (10E3/µL)**	6.3 (2.2)	6.1 (1.5)	6.9 (1.7)	0.06	5.8 (2.6)	6.4 (2.9)	0.28	0.12	**0.03**	0.77
**Lymphocytes (10E3/µL)**	2.0 (2.3)	1.8 (0.5)	2.5 (3.7)	0.24	1.6 (0.6)	1.5 (0.6)	0.72	0.07	0.16	0.34
**Neutrophils (10E3/microL)**	3.86 (1.77)	3.6 (1.2)	4.2 (1.1)	**0.04**	3.5 (2.3)	4.2 (2.4)	0.14	0.54	0.06	0.87
**Neutrophils/lymphocytes ratio**	2.6 (2.6)	2.1 (0.8)	2.2 (0.8)	0.46	2.6 (2.3)	4.0 (5.7)	0.10	0.07	0.49	0.18
**Monocytes (10E3/microL)**	0.51 (0.81)	0.3 (0.1)	0.8 (1.3)	**<0.001**	0.4 (0.2)	0.4 (0.2)	0.30	0.12	**0.04**	0.20
**Platelets (10E3/µL)**	236.6 (57.7)	233.71(43.7)	240.0 (51.1)	0.75	234.1 (64.3)	237.9 (77.6)	0.72	0.81	0.60	0.92
**Hemoglobin (g/dL)**	14.65 (1.25)	15.0 (1.5)	15.0 (1.3)	0.73	14.2 (1.0)	14.3 (1.0)	0.74	**<0.001**	0.12	0.80
**Hematocrit (%)**	44.0 (3.65)	45.3 (4.4)	45.0 (3.7)	0.79	42.5 (2.6)	42.8 (3.1)	0.81	**<0.001**	0.22	0.66
**aPTT (s)**	31.8 (6.6)	29.1 (2.3)	30.4 (4.0)	**0.02**	34.2 (7.1)	34.1 (11.4)	0.21	**<0.001**	0.56	0.54
**RDW (%)**	13.9 (2.2)	13.7 (0.7)	13.6 (0.8)	0.57	13.7 (1.1)	15.4 (5.1)	0.13	0.13	0.25	**0.03**
**Fibrinogen (mg/dL)**	361.3 (95.8)	359.5 (95.1)	366.8 (76.4)	0.38	325.8 (79.2)	427.7 (135.3)	**0.002**	0.73	**0.01**	**<0.01**
**D-dimer (ng/mL)**	333.2 (162.8)	318.2 (137.1)	329.2 (140.3)	0.71	307.0 (156.6)	422.5 (232.9)	0.16	0.57	0.16	0.12
**C-reactive protein (mg/L)**	4.5 (7.3)	2.5 (2.7)	4.6 (4.3)	**0.02**	3.6 (9.7)	8.6 (10.1)	**0.003**	0.31	**0.05**	0.31
**IL-6 (pg/mL)**	3.6 (1.9)	3.7 (2.6)	3.5 (1.6)	0.30	3.5 (2.0)	3.7 (1.5)	0.15	0.95	0.90	0.65
**Ferritin (ng/mL)**	109.2 (87.0)	151.4 (96.7)	120.0 (93.3)	0.18	87.0 (77.0)	75.8 (49.3)	0.83	**<0.01**	0.75	0.54
**NT proBNP (pg/mL)**	78.0 (64.6)	75.7 (65.2)	82.2 (81.1)	0.89	62.8 (32.5)	101.9 (65.7)	0.04	0.75	0.10	0.20

Data presented as mean (x), standard deviation (SD), and *p* values. The significance threshold was set at p < 0.05*. *P* value refers to the comparison of variables’ mean between patients with low-grade MI and patients with SLE using t-test or Mann-Whitney test, according to the distribution of the data. Low BMI refers to BMI ≤ 30 kg/m^2^ and high BMI refers to BMI ≥ 30 kg/m^2^. “Group of disease” column is the comparison of variables’ mean between the two inflammatory diseases (without considering BMI status). “BMI categories” column is the comparison of variables’ mean between the low and high BMI. Interaction column means the *P* value of comparison between the two disease groups and the BMI categories (ANOVA 2X2). aPTT, Activated Partial Thromboplastin Time (s, seconds); IL-6, Interleukin-6; NT proBNP, Natriuretic Peptide Tests; RDW, Red Cell Blood Distribution Width. *P* value lower than 0.05 in bold type.

Finally, significant interactions between BMI categories and disease status were detected for body fat, body water, and waist circumference, highlighting that the influence of disease on body composition is modulated by BMI category.

Table 2 shows the comparisons of hematological and inflammatory markers between type of disease and stratified by BMI. I

Participants with low-grade MI and higher BMI exhibited increased markers of systemic inflammation and coagulation, including neutrophils (key innate immune cells), monocytes (circulating precursors of macrophages and dendritic cells), aPTT (activated partial thromboplastin time, a marker of coagulation activity), and CRP (Table 1), suggesting an enhanced inflammatory and pro-thrombotic profile (Table 2).

As shown in Table 2, individuals with high BMI also showed higher monocyte counts, and SLE patients with high BMI showed elevated leukocyte levels, supporting the link between BMI and systemic inflammation. SLE participants with high BMI showed higher fibrinogen and CRP levels, consistent with an amplified pro-inflammatory and pro-thrombotic profile in the context of excess of body weight. When comparing BMI categories irrespective of disease status (Table 2), both biomarkers also showed significant differences, highlighting the connection between BMI and systemic inflammatory processes. Moreover, the interaction analysis revealed that RDW and fibrinogen were jointly influenced by disease type and BMI, suggesting that body weight may modulate hematological and coagulation alterations in SLE.

### Analysis of gut microbiota community profiling according to type of inflammatory disease and BMI stratification: alpha and beta gut diversity

**Fig 1:**
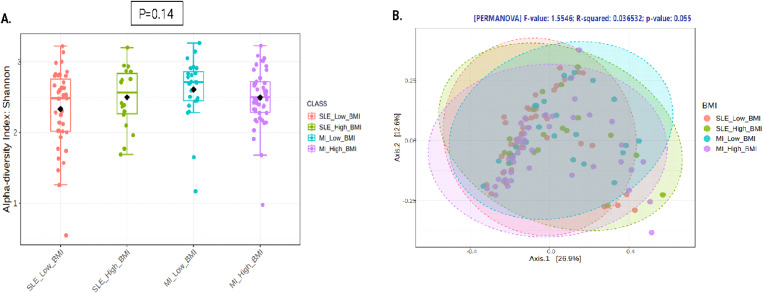
Alpha diversity analysis evaluated by diversity measure using Shannon index for genus level according to the type of disease and BMI status. Red boxes represent patients with SLE and low BMI; green boxes represent patients with SLE and high BMI; light blue boxes represent patients with low-grade MI and low BMI and purple boxes represent patients with low-grade MI and high BMI (A). Principal coordinate analysis for beta diversity calculated using Bray Curtis index and PERMANOVA test for genus. Red circles represent patients with SLE and low BMI; green boxes represent patients with SLE and high BMI; light blue boxes represent patients with low-grade MI and low BMI and purple boxes represent patients with low-grade MI and high BMI (B).

The analysis of alpha diversity evaluated using the Shannon index showed no significant differences (*p* = 0.14) between groups in this population (Fig 1A). Similarly, the analysis of beta diversity using the Bray-Curtis distance method revealed no significant differences (*p* = 0.055) (Fig 1B), although the p value was 0.055.

### Analysis of gut microbiota composition according to type of inflammatory disease and BMI stratification: searching of biomarkers and analysis of differential abundance

**Fig 2:**
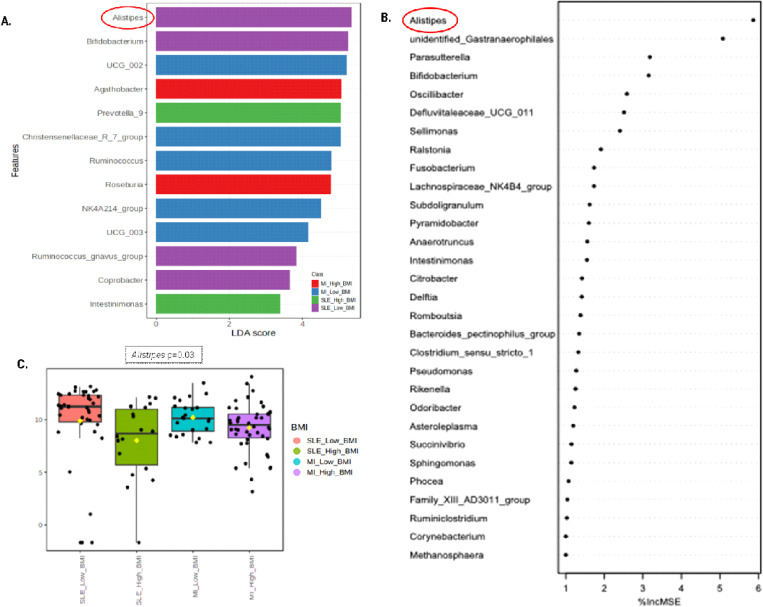
The analysis of the microbial structure based on linear discriminant analysis (A) and Random Forest (B), abundance analysis using EdgeR (C) according to the type of inflammatory disease and body weight in METAINFLAMMATION cohort. The most differentially abundant taxa between patients with low-grade MI and SLE according to BMI are represented in a bar graph LDA score, an estimation of the effect size. Only taxa meeting a *P* < 0.05 and LDA score significant threshold | > 2| are shown. Red, bacterial taxa statistically overrepresented in low-grade MI participants and high BMI; blue, bacterial taxa overrepresented in participants with low-grade MI and low BMI; green, bacterial taxa overrepresented in participants with SLE and high BMI and purple bacterial taxa overrepresented in participants with SLE and low BMI (A).The Random Forest analysis was performed with the importance plot showing the importance of each variable in the prediction of the model, where the first variable is the most important when discerning between a low and a high BMI (B). The differential abundance analysis performed using EdgeR between patients with low-grade MI and SLE are represented with a bar plot (*P* value corrected by FDR). Red boxes represent patients with SLE and low BMI; green boxes represent patients with SLE and high BMI; light blue boxes represent patients with low-grade MI and low BMI and purple boxes represent patients with low-grade MI and high BMI (C).

A comparative analysis of the taxonomic structure of the gut microbiota was conducted to offer an in-depth understanding and to identify significant patterns, variances, and relationships among the analyzed groups. Linear discriminant analysis showed the differential abundance analysis performed to identify genus significantly different between low-grade MI and SLE patients and low and high BMI (Fig 2A). Moreover, the Random Forest analysis showed that the genus *Alistipes* is the most important genera to distinguish between low and high BMI, as shown in the importance plot (Fig 2B). The analysis of differential abundance with EdgeR method showed that *Alistipes* genus was the most abundant bacteria in participants with SLE and low BMI, with a p value of 0.03 (Fig 2C). These analyses show that *Alistipes* presented a differential abundance depending on the type of inflammatory disease and the BMI stratification. In this analysis, differential abundance was assessed across the four predefined subgroups (SLE low BMI, SLE high BMI, MI low BMI, MI high BMI). The aim of Fig 2 is to illustrate how *Alistipes* emerged at the genus level as a taxonomic feature of interest when considering both disease group and BMI stratification.

### Relationship between inflammatory condition, BMI stratification and *Alistipes*

**Fig 3:**
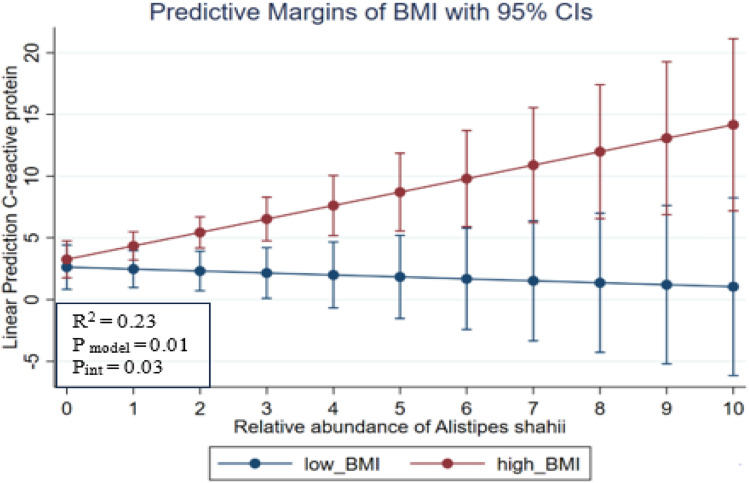
Predicted values of C-reactive protein in patients with low BMI and high BMI, according to the relative abundance of *Alistipes shahii* patients with SLE (R^2^ = 0.23; *P* value of the interaction = 0.03). Blue line represents values for patients with low BMI (≤ 30 kg/m^2^) and the red line represents values for high BMI (≥30 kg/m^2^). The model was adjusted for age, sex, and adherence to Mediterranean diet.

Considering that CRP presented significant differences between groups of comparisons, a regression analysis was performed to explore the potential interaction between CRP (as dependent variable) Alistipes spp. abundance levels (as independent variable) and BMI status as low BMI and high BMI. A significant interaction was found between the mentioned variables in patients with SLE (Fig 3) (R2 = 0.23; P value of the interaction = 0.03), showing that participants with high BMI (red line in Fig 3) and higher abundance of *Alistipes shahii* presented higher predicted values of CRP. However, participants with higher values of *Alistipes shahii* but low BMI (blue line in Fig 3) showed lower values of CRP, suggesting an effect modification of *Alistipes shahii* on CRP depending on BMI. No significant results were found for low-grade MI individuals.

Additionally, a regression model was constructed using CRP as a dependent variable (adjusted by age, sex, disease) and the interaction between *Alistipes, Alistipes onderdonkii, Alistipes shahii, Alistipes obesi*, and BMI respectively as independent variables. The first model with Alistipes did not evidence significant changes, but with *Alistipes onderdonkii* an increase of one unit in BMI was associated with an average increase of 0.50 units in CRP (P < 0.001). Also, an increase of one unit of *Alistipes onderdonkii* showed an average decrease of 2.55 units in CRP (*p* = 0.04), while the interaction between *Alistipes onderdonkii* and BMI showed an increase in 0.09 units in CRP (*p* = 0.02). On the other hand, the positive coefficients of 0.34 and 0.16 for the BMI and the interaction between *Alistipes shahii* and BMI evidenced higher values of CRP (*p* = 0.01) respectively. In addition, the increase of one unit in *Alistipes shahii* was associated with a decrease of 4.73 units in CRP (*P* = 0.02). Likewise, the gender and BMI also showed variations in their regression coefficients with an increase of 3.00 units (p = 0.02) and 0.58 units (p < 0.001) of CRP respectively (Supplementary S2 Table).

Also, the other models adjusted by inflammatory markers evidenced significant changes, when adjusted by leukocytes as an inflammatory marker the interaction between *Alistipes* and BMI categories increase of one unit was associated with an average increase of 1.45 units for CRP (*P* = 0.03) while when adjusted by NLR the interaction between *Alistipes* and BMI categories showed an increase of 1.36 units in CRP (*p* = 0.04).

On the other hand, the positive coefficient of 1.43 for the interaction between *Alistipes* genus and BMI categories when adjusted by lymphocytes showed higher values of CRP (*p* = 0.03), in addition the increase of the interaction between *Alistipes* and BMI categories was associated with an increase of 1.32 (*P* = 0.04) and 0.84 units (*P* = 0.02) respectively for CRP when adjusted by IL-6 (Supplementary S3 Table).

**Fig 4:**
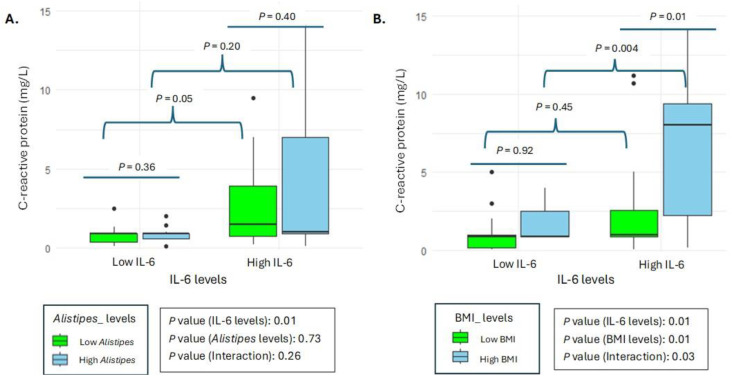
Differences in the C-reactive protein between individuals with different categories of *Alistipes* within each group IL-6 and different categories of BMI within each group of IL-6, in participants with SLE. Green boxes represent low abundance of *Alistipes,* and blue boxes represent high abundance of *Alistipes* (A). Relationship between BMI categories and C-reactive protein in patients with SLE. Green boxes represent participants with low BMI and blue boxes represent high BMI (B).

Furthermore, the analysis in Fig 4 was performed to compare CRP levels among individuals with SLE according to BMI categories and *Alistipes* levels. This analysis was conducted to investigate possible associations between CRP-measured inflammation, BMI and *Alistipes* abundance in the microbiota of patients. Fig 4A showed a box plot representing CRP concentration, distinguishing between two categories based on *Alistipes* levels: low *Alistipes* and high *Alistipes*. Additionally, the boxes were colored to differentiate between two categories of IL-6 (low and high) in patients with SLE.

The results indicated that there were no statistically significant differences in CRP levels between the different groups based on IL-6 and *Alistipes* levels (*p* = 0.26 for the interaction). However, when analyzing the effects of IL-6 and *Alistipes* individually, significant differences in CRP levels were found. Specifically, a significant association was observed between IL-6 levels and CRP (*P* = 0.01), suggesting an influence of systemic inflammation on inflammatory markers.

On the other hand, no significant differences in CRP levels were found between the low and high *Alistipes* groups (*p* = 0.73), suggesting that *Alistipes* levels might not be directly related to systemic inflammation in SLE patients.

Also, the Fig 4B showed a box plot representing CRP concentration, distinguishing between two categories based on BMI categories: low BMI and high BMI. Additionally, the boxes were colored to differentiate between two categories of IL-6 (low and high) in patients with SLE. The results revealed statistically significant differences in CRP levels between the different groups based on IL-6 and BMI categories (*P* = 0.03 for interaction), indicating a significant interaction between these factors.

Breaking down the individual effects, a significant association was observed between IL-6 levels and CRP levels (*P* = 0.01), suggesting that elevated IL-6 levels are associated with an increase in CRP concentration, a key marker of inflammation. This reinforces the idea that systemic inflammation, represented by high levels of IL-6, has a considerable impact on CRP levels in patients with SLE.

Likewise, BMI categories also showed a significant association with CRP (*p* = 0.01), indicating that patients with a high BMI tend to have higher CRP levels. This finding suggests that BMI may contribute to a more pronounced inflammatory state in these patients.

The significant interaction between IL-6 and BMI categories (p* *= 0.03) suggests that the combined effect of these factors on CRP levels is greater than the sum of their individual effects. In other words, the influence of elevated IL-6 levels on CRP levels is amplified in patients with high BMI.

## Discussion

This study investigated the impact of BMI on diverse anthropometric, biochemical and inflammatory characteristics in patients with low-grade MI and SLE, using a 2x2 factorial design. In addition, both conditions are influenced by the composition and functionality of gut microbiota, which in turn modulates systemic inflammation and body weight regulation [17]

Individuals with MI and high BMI showed higher values for all anthropometric and body composition variables, except for body water. These findings indicate that BMI affects these variables differently depending on the presence of the type of inflammatory disease (low-grade MI or SLE). The scientific literature largely confirms that people with a higher BMI tend to have worse values for anthropometric and body composition measures as well as biochemical values [39,40]. A significant interaction was found for body fat percentage, waist circumference, BMI and body water, indicating a differential effect according to the type of inflammatory disease and BMI. These findings suggest that the relationship between body weigh and systemic inflammation may not be uniform across different inflammatory disease profiles [41,42]. Instead, they point toward disease-specific modulation of adiposity-related metabolic alterations, in which both the type of inflammatory disease and the BMI category interact to influence these parameters [43]. This differential effect may reflect distinct pathophysiological mechanisms underlying fat distribution, fluid balance, and metabolic activity in individuals with chronic inflammation, emphasizing the need for tailored therapeutic strategies based on disease phenotype and nutritional status [44]. It is important to note that the interaction between BMI and disease group for adiposity-related parameters, such as body fat percentage and waist circumference, should be interpreted with caution. Patients with metabolic inflammation typically present with higher baseline BMI, which may limit further increases in adiposity per unit BMI. In contrast, patients with SLE generally start at lower BMI levels, potentially amplifying the slopes observed in regression models. Therefore, at least part of the interaction effects may reflect underlying baseline disparities rather than distinct biological mechanisms. Supplementary S1 Table presents descriptive statistics for relevant metabolic and inflammatory parameters separately by group (Supplementary S1 Table), which enables readers to better contextualize these results.

Regarding the biochemical profile, SLE-high BMI patients had greater levels of triglycerides, glycosylated hemoglobin, and insulin. These findings are consistent with research that found a 25.6% prevalence of metabolic syndrome, directly attributed to high BMI, and showed a predisposition to insulin resistance and chronic inflammation concurrent to the disease [45,46]. This underlines the importance of monitoring body weight and body composition in SLE patients as essential for comprehensive management and accurate risk assessment [47].

In the analysis of hematological parameters and inflammatory markers, it was found that patients with MI-high BMI showed higher levels of neutrophils, monocytes and activated partial thromboplastin time (aPTT) and CRP. This finding is in line with previous studies highlighting the relevance of these elements in obesity-related inflammation and metabolic syndrome [48]. Patients with SLE-higher BMI had significantly elevated levels of fibrinogen and CRP, suggesting increased inflammatory and clotting activity, as evidenced in other studies [49–51]. Fibrinogen, extensively studied in SLE, is associated with cardiovascular risk [52]. In addition, it plays a crucial role in inflammation and coagulation, processes that are dysregulated in this disease [49]. CRP levels are significantly associated with cardiovascular risk and SLE. Several studies have shown that patients with SLE have higher CRP levels, indicating an increased cardiovascular risk [53]. Elevated CRP levels in SLE patients are associated with cardiometabolic risk factors and clinical disease activity, highlighting the role of inflammation in the development of cardiovascular disease [54,55]. Therefore, the European League Against Rheumatism (EULAR) has generated recommendations for managing cardiovascular risk in these patients [56]. However, CRP levels may fluctuate over time and are influenced by factors such as age, menopausal status, smoking and infections [57]. This suggests that CRP plays a complex role in the inflammatory processes of SLE, affecting disease activity and reflecting various immunological effects. A significant interaction was found between disease type and BMI in relation to fibrinogen and RDW, indicating that both factors jointly influence these hematological markers [58,59]. These findings further support the notion that elevated BMI adversely influences both inflammatory and coagulation pathways [60]. In addition, statistical significance was evident in the disease groups in relation to hemoglobin and hematocrit.

Linear discriminant analysis and Random Forest revealed that the genus *Alistipes* was key in patients with SLE and low BMI, suggesting a possible role in the interaction between inflammatory disease type and BMI [61], as this bacterial genus is involved in inflammatory responses and metabolite production. Some research suggests that it may be pathogenic in colorectal cancer whose presence may be affected by gut dysbiosis [62]. However, other studies indicate that *Alistipes* plays an important role in BMI regulation and body weight maintenance [61]. Although a direct link between *Alistipes* and SLE has not been established, understanding how the gut microbiome influences inflammatory diseases opens new opportunities to explore possible connections.

The interaction observed in this study between the relative abundance of *Alistipes* and BMI on CRP levels in SLE patients suggests that gut microbiota composition and BMI together have a significant impact on CRP levels. These findings suggest the importance of considering the interaction between different inflammatory markers in the pathogenesis of SLE and highlight the need for additional research to better understand the underlying mechanisms. In subjects with higher BMI, as *Alistipes* increases, CRP concentration also increases. This finding could allow for more personalized and precise interventions, promoting improved health status. In this case, a direct proportional association was found between BMI levels and CRP, as well as between *Alistipes shahii* levels and CRP. Specifically, higher BMI and higher *Alistipes shahii* abundance together generate a significant change in CRP levels. Several studies have shown that elevated BMI is associated with higher CRP levels, indicating that obesity contributes to systemic inflammation through the release of inflammatory mediators by adipose tissue [63]. Furthermore, the relationship between *Alistipes* and CRP levels may be of interest due to the possible involvement in the regulation of inflammation. *Alistipes* has also been associated with healthier metabolic profiles and a role in body weight regulation. However, other studies show an increase in this genus in people with obesity [64,65]. Particularly, *Alistipes shahii*, belonging to the phylum Bacteroidetes, has been investigated for its potential in the prevention of metabolic diseases such as atherosclerosis and obesity [61,66]. These findings underline the importance of considering the composition of the gut microbiota and controlling body weight to reduce inflammation and lower the risk of associated chronic diseases. Both factors have a joint impact on inflammation, which could allow for more personalized and effective interventions to improve health status. In this sense, species-level analyses of *Alistipes* (*A. onderdonkii*, *A. shahii*, *A. obesi*) revealed strain-specific associations with BMI and systemic inflammation. While *A. onderdonkii* correlates with IL-6 and CRP in obese individuals [67], other strains may influence lipid metabolism differently [68]. These findings highlight that not all *Alistipes* species contribute equally to metabolic or inflammatory processes, providing a mechanistic explanation for BMI- and disease-specific differences observed in our cohort.

In patients with systemic lupus erythematosus (SLE), elevated CRP levels are not solely attributable to BMI but also reflect chronic systemic inflammation driven by autoimmune dysregulation, contributing to increased cardiovascular risk independent of traditional factors such as hypertension or dyslipidemia (Pesqueda-Cendejas et al., 2022, [link pending DOI]). Alterations in gut microbiota composition, including reduced abundance of genera such as *Alistipes*, may interact with this autoimmune environment, potentially modulating inflammatory markers like CRP and IL-6 differently than in individuals with low-grade metabolic inflammation [69]Dysbiosis in SLE has been associated with increased intestinal permeability, altered immune signaling, and systemic inflammation, highlighting disease-specific effects on the gut–systemic inflammation axis.

Moreover, inflammatory adipokines and cytokines may exhibit altered dynamics in SLE due to persistent immune activation, further influencing systemic inflammatory responses and metabolic pathways [70]. These disease-specific interactions underscore the importance of integrating microbiota composition, body composition, and inflammatory profiling to inform more precise cardiovascular risk assessment and to guide personalized therapeutic strategies in SLE.

As expected, the box plot shows that higher CRP is associated with elevated IL-6 concentrations with respect to *Alistipes* abundance and BMI. Analysis of the data through the box plot reveals a clear association between elevated CRP levels and elevated IL-6 concentrations in relation to *Alistipes* abundance and BMI. In particular, the results highlight that high circulating levels of IL-6 interact with BMI significantly, exacerbating CRP values in individuals with high BMI compared to those with lower BMI. This interaction suggests that systemic inflammation as measured by CRP is not only influenced by the presence of IL-6 but is also exacerbated in the context of obesity. The direct proportional relationship between BMI and CRP levels indicates that as BMI increases, so do CRP levels, indicating a more pronounced inflammatory state in individuals with higher BMI. This finding is consistent with previous studies that have shown that obesity is associated with a chronic inflammatory state [71], which can be measured through biomarkers such as CRP [72]. Furthermore, the observation that lower IL-6 levels correlate with lower CRP levels reinforces the hypothesis that IL-6 plays a crucial role in mediating systemic inflammation [73,74]. In contrast, in the group with higher IL-6 levels, CRP levels increase significantly, underlining the importance of IL-6 as an inflammatory modulator in individuals with higher BMI [74]. This phenomenon may be explained by the increased production of IL-6 in adipose tissue, which in turn contributes to a higher plasma CRP concentration [75]. Increased IL-6 in individuals with higher BMI leads to a disproportionate increase in CRP, highlighting a synergistic interaction that exacerbates systemic inflammation. This finding contrasts with the interaction between *Alistipes* and IL-6, which does not show comparable statistical significance. This suggests that, although *Alistipes* may influence CRP levels, its effect is not as pronounced as that of IL-6 in the context of elevated BMI. One possible explanation for the observed association between *Alistipes* abundance and elevated CRP in lupus patients with higher BMI is that certain species of *Alistipes* may produce pro-inflammatory metabolites, such as lipopolysaccharides or short-chain fatty acids that modulate immune responses. Additionally, altered gut barrier integrity associated with dysbiosis could increase systemic exposure to microbial components, thereby promoting inflammation. In individuals with higher BMI, interactions between gut-derived inflammatory signals and adipose tissue–derived cytokines may further amplify CRP production. Supporting this notion, a recent study in humans reported that *Alistipes* species, such as *Alistipes shahii*, are enriched in patients with SLE and may contribute to a pro-inflammatory microbial profile [76]. While these mechanisms remain speculative and require experimental validation, they provide a biologically plausible context for our findings and suggest potential avenues for future research into microbiota-mediated modulation of inflammation in SLE. In any case, a novel insight from our study is the role of BMI as an effect modifier in the IL-6–CRP axis within SLE patients. Stratification of IL-6–CRP by low versus high BMI, as shown in Fig 4B, highlights that participants with higher BMI exhibit a steeper IL-6–CRP gradient, suggesting that BMI amplifies systemic inflammation beyond the autoimmune component alone. While the overall correlation between IL-6 and CRP is well-known, few studies have visualized how BMI modulates this relationship in an autoimmune context, making this representation a useful framework for interpreting patient-specific inflammatory burden [77]. In contrast, *Alistipes* abundance does not show comparable statistical significance, indicating that microbial contributions may emerge more subtly or at the species level.

Nevertheless, this research presents some limitations. The sample size is relatively small, but a detailed comparison of body composition, biochemical profiles, and inflammatory markers between patients with low-grade MI and SLE, stratified by BMI, is provided allowing a more precise understanding of the interaction between these factors and the type of inflammatory disease. In any case, despite that type I y II errors cannot be discarded, the observed outcomes are plausible with existing related evidence. Although imaging-based measures of visceral adiposity were not available, bioimpedance-derived indices (visceral fat AU, body fat percentage, and waist circumference) were included and complemented with circulating inflammatory markers (CRP and IL-6), both closely related to visceral adipose deposition. Moreover, recent evidence supports the use of bioimpedance-derived visceral fat estimates, which have been shown to be independently associated with inflammatory biomarkers such as CRP and IL-6 [78,79].

In summary, the differences in body composition and biochemical profiles between patients with low-grade MI and SLE indicate the need for putative differentiated diagnosis and therapeutic approaches. CRP levels are affected by BMI and *Alistipes shahii* abundance in lupus patients, but as *Alistipes shahii* level increases, CRP concentration substantially increases more in subjects with high BMI. In addition, higher CRP is associated with elevated IL-6 concentrations with respect to *Alistipes* abundance and BMI. Importantly, IL-6 showed elevated circulating levels and interaction with BMI since that CRP values were exacerbated in the high BMI groups compared to those with lower BMI. This investigation highlights the importance of not only including microbiota analysis in inflammatory diseases management, but also of conjointly considering treatments based on body weight and inflammatory markers to provide a more personalized and effective approach in lupus.

## Conclusion

This research corroborated that excess body weight significantly affects anthropometric and biochemical variables and inflammatory markers in patients with low-grade MI and SLE. Our results indicate that CRP levels are influenced by BMI and the relative abundance of *Alistipes shahii*. Interesting, in subjects with a higher BMI, an increase in *Alistipes* abundance is associated with an exacerbated CRP concentration. In addition, elevated IL-6 levels interact significantly with BMI, elevating CRP values in individuals with high BMI compared to those with lower BMI. These findings have important implications for understanding the underlying mechanisms of inflammation in obesity and SLE and may guide future research towards interventions that modulate IL-6 as a strategy to reduce systemic inflammation in high-BMI individuals. These results also reinforce the need to consider BMI and gut microbiota as modulatory factors in managing inflammatory diseases.

## Supporting information

S1 Table Key metabolic and inflammatory parameters in Low-grade Metabolic Inflammation (LMI) and Systemic Lupus Erythematosus (SLE) participants in the METAINFLAMMATION cohort. (DOCX)

S2 Table Multiple regression models for the CRP adjusted for age, sex, disease, and the interaction between *Alistipes*, *Alistipes onderdonkii*, *Alistipes shahii*, *Alistipes obesi*, and BMI respectively in the METAINFLAMMATION cohort. (DOCX)

S3 Table Multiple regression models for the CRP adjusted for age, sex, disease, inflammatory markers (leukocytes, NLR, lymphocytes and IL-6,) and the interaction between *Alistipes* and BMI categories in the METAINFLAMMATION cohort. (DOCX)

S1 Fig Flowchart of METAINFLAMATION project participants. (PDF)
