# Supplementary material for: Body mass index distinctly modulates the associations between Alistipes and CRP/IL-6 in metabolic and lupus inflammatory features
Source: PLoS One. 2025 Nov 25;20(11):e0335452. doi: 10.1371/journal.pone.0335452 (PMC12646403; doi:10.1371/journal.pone.0335452)
Supplement: S1 Table — (DOCX) [file pone.0335452.s001.docx]

|  | Low-grade Metabolic Inflammation (n= 70) | Systemic Lupus Erythematosus (n= 57) | P value |
| --- | --- | --- | --- |
| BMI (kg/m²) | 31.9 (4.1) | 28.0 (5.6) | <0.001 |
| Body fat (%) | 37.5 (6.8) | 35.3 (8.7) | 0.12 |
| Waist circumference (cm) | 110.3 (10.9) | 96.8 (13.7) | <0.001 |
| CRP (mg/L) | 3.9 (3.9) | 5.2 (10.0) | 0.35 |
| IL-6 (pg/mL) | 3.6 (1.9) | 3.5 (1.8) | 0.71 |

Data are presented as mean (standard deviation). P values correspond to comparisons between groups using independent t-tests.
