# Supplementary material for: Body mass index distinctly modulates the associations between Alistipes and CRP/IL-6 in metabolic and lupus inflammatory features
Source: PLoS One. 2025 Nov 25;20(11):e0335452. doi: 10.1371/journal.pone.0335452 (PMC12646403; doi:10.1371/journal.pone.0335452)
Supplement: S2 Table — (DOCX) [file pone.0335452.s002.docx]

|  |  | **Dependent variable: CRP** | **CRP** | | ***Alistipes*** | |  | | | ***Alistipes onderdonkii*** | |  |  | | ***Alistipes shahii*** | | |  |  | | ***Alistipes obesi*** | | |  | |  |
| --- | --- | --- | --- | --- | --- | --- | --- | --- | --- | --- | --- | --- | --- | --- | --- | --- | --- | --- | --- | --- | --- | --- | --- | --- | --- | --- |
|  |  | | |  | | **R squared=0.33** | |  |  | | **R squared=0.36** | | |  | |  | **R squared=0.35** | | |  | |  | **R squared=0.34** | |  | |
|  |  | | |  | |  | |  |  | |  | | |  | |  |  | | |  | |  |  | |  | |
|  | **MULTIPLE REGRESSION** | | | **Regression coefficient (β)** | | **CI 95%** | | **P** | **Regression coefficient (β)** | | **CI 95%** | | | **P** | | **Regression coefficient (β)** | **CI 95%** | | | **P** | | **Regression coefficient (β)** | **CI 95%** | | **P** | |
|  | **Intercept** | | | -8.62 | | -23.39 to 6.16 | | 0.25 | -11.89 | | -20.28 to -3.49 | | | 0.01 | | -5.98 | -16.43 to 4.46 | | | 0.26 | | -14.66 | -23.12 to -6.19 | | 0.001 | |
|  | **Age** | | | -0.02 | | -0.11 to 0.07 | | 0.63 | -0.02 | | -0.11 to 0.06 | | | 0.58 | | -0.04 | -0.14 to 0.05 | | | 0.34 | | -0.02 | -0.11 to 0.07 | | 0.65 | |
|  | **Gender** | | | 2.15 | | -0.42 to 4.72 | | 0.10 | 1.95 | | -0.54 to 4.44 | | | 0.12 | | 1.83 | -0.82 to 4.48 | | | 0.17 | | 3.00 | 0.50 to 5.50 | | **0.02** | |
|  | **Disease** | | | 1.59 | | -0.73 to 3.91 | | 0.18 | 1.07 | | -1.20 to 3.34 | | | 0.35 | | 1.20 | -1.04 to 3.45 | | | 0.29 | | 1.14 | -1.12 to 3.40 | | 0.32 | |
|  | **BMI** | | | 0.35 | | -0.09 to 0.79 | | 0.12 | 0.50 | | 0.31 to 0.69 | | | **<0.001** | | 0.34 | 0.08 to 0.59 | | | **0.01** | | 0.58 | 0.39 to 0.77 | | **<0.001** | |
|  | ***Alistipes*** | | | -1.71 | | -5.37 to 1.94 | | 0.35 |  | |  | | |  | |  |  | | |  | |  |  | |  | |
|  | ***Alistipes*: BMI** | | | 0.07 | | -0.05 to 0.19 | | 0.25 |  | |  | | |  | |  |  | | |  | |  |  | |  | |
|  | ***Alistipes onderdonkii*** | | |  | |  | |  | -2.55 | | -4.92 to -0.18 | | | **0.04** | |  |  | | |  | |  |  | |  | |
|  | ***Alistipes onderdonkii*: BMI** | | |  | |  | |  | 0.09 | | 0.02 to 0.17 | | | **0.02** | |  |  | | |  | |  |  | |  | |
|  | ***Alistipes shahii*** | | |  | |  | |  |  | |  | | |  | | -4.73 | -8.51 to -0.94 | | | **0.02** | |  |  | |  | |
|  | ***Alistipes shahii*: BMI** | | |  | |  | |  |  | |  | | |  | | 0.16 | 0.04 to 0.29 | | | **0.01** | |  |  | |  | |
|  | ***Alistipes obesi*** | | |  | |  | |  |  | |  | | |  | |  |  | | |  | | 3.07 | -0.92 to 7.06 | | 0.13 | |
| ***Alistipes obesi*: BMI** | | | |  | |  | |  |  | |  | | |  | |  |  | | |  | | -0.12 | -0.24 to 0.01 | | 0.08 | |

Data was presented as regression coefficient(β), confidence intervals (95%) and *P* values. Adjusted for age, age, sex, disease, and the interaction between *Alistipes*, *Alistipes onderdonkii*, *Alistipes shahii*, *Alistipes obesi*, and BMI respectively in the METAINFLAMMATION cohort. BMI, Body Mass Index.
