# Supplementary material for: Body mass index distinctly modulates the associations between Alistipes and CRP/IL-6 in metabolic and lupus inflammatory features
Source: PLoS One. 2025 Nov 25;20(11):e0335452. doi: 10.1371/journal.pone.0335452 (PMC12646403; doi:10.1371/journal.pone.0335452)
Supplement: S3 Table — (DOCX) [file pone.0335452.s003.docx]

|  |  | **Dependent variable: CRP** | **CRP** | | **Leukocytes** | |  | | | **NLR** | |  |  | | **Lymphocytes** | | |  |  | | **IL-6** | | |  | |  |
| --- | --- | --- | --- | --- | --- | --- | --- | --- | --- | --- | --- | --- | --- | --- | --- | --- | --- | --- | --- | --- | --- | --- | --- | --- | --- | --- |
|  |  | | |  | | **R squared=0.24** | |  |  | | **R squared=0.25** | | |  | |  | **R squared=0.23** | | |  | |  | **R squared=0.27** | |  | |
|  |  | | |  | |  | |  |  | |  | | |  | |  |  | | |  | |  |  | |  | |
|  | **MULTIPLE REGRESSION** | | | **Regression coefficient (β)** | | **CI 95%** | | **P** | **Regression coefficient (β)** | | **CI 95%** | | | **P** | | **Regression coefficient (β)** | **CI 95%** | | | **P** | | **Regression coefficient (β)** | **CI 95%** | | **P** | |
|  | **Intercept** | | | 1.23 | | -6.72 to 9.18 | | 0.76 | 2.34 | | -4.84 to 9.52 | | | 0.52 | | 4.21 | -2.80 to 11.23 | | | 0.24 | | 1.85 | -5.20 to 8.90 | | 0.60 | |
|  | **Age** | | | -0.03 | | -0.13 0.07 | | 0.56 | -0.04 | | -0.13 to 0.06 | | | 0.48 | | -0.04 | -0.04 to 0.06 | | | 0.40 | | -0.06 | -0.15 to 0.04 | | 0.24 | |
|  | **Gender** | | | 1.87 | | -0.90 to 4.64 | | 0.18 | 1.97 | | -0.79 to 4.73 | | | 0.16 | | 1.59 | -1.22 to 4.40 | | | 0.26 | | 1.67 | -1.04 to 4.39 | | 0.22 | |
|  | **Disease** | | | 1.21 | | -1.26 to 3.68 | | 0.33 | 0.58 | | -1.88 to 3.04 | | | 0.64 | | 0.88 | -1.58 to 3.34 | | | 0.48 | | 0.79 | -1.61 to 3.18 | | 0.52 | |
|  | ***Alistipes*** | | | -0.42 | | -1.38 to 0.53 | | 0.38 | -0.37 | | -1.30 to 0.57 | | | 0.44 | | -0.31 | -1.26 to 0.65 | | | 0.52 | | -0.25 | -1.18 to 0.68 | | 0.59 | |
|  | **BMI_ levels High BMI** | | | -0.43 | | -5.07 to 4.22 | | 0.86 | 0.11 | | -4.45 to 4.67 | | | 0.96 | | 0.22 | -4.40 to 4.84 | | | 0.93 | | 0.29 | -4.22 to 4.79 | | 0.90 | |
|  | ***Alistipes*: BMI_levels High BMI** | | | 1.45 | | 0.15 to 2.75 | | **0.03** | 1.36 | | 0.07 to 2.66 | | | **0.04** | | 1.43 | 0.12 to 2.74 | | | **0.03** | | 1.32 | 0.04 to 2.59 | | **0.04** | |
|  | **Leukocytes** | | | **0.35** | | 0.98 to 1.01 | | 0.16 |  | |  | | |  | |  |  | | |  | |  |  | |  | |
|  | **NLR** | | |  | |  | |  | 0.58 | | -0.11 to 1.27 | | | 0.10 | |  |  | | |  | |  |  | |  | |
|  | **Lymphocytes** | | |  | |  | |  |  | |  | | |  | | -0.17 | -0.60 to 0.26 | | | 0.42 | |  |  | |  | |
|  | **IL-6** | | |  | |  | |  |  | |  | | |  | |  |  | | |  | | 0.84 | 0.12 to 1.56 | | **0.02** | |

Data was presented as regression coefficient (β), confidence intervals (95%) and *P* values. Adjusted for age, sex, disease, leukocytes, NLR, lymphocytes, IL-6, and the interaction between *Alistipes* and BMI categories in the METAINFLAMMATION cohort. BMI, Body Mass Index; NLR, IL-6, Interleukin-6; NLR, Neutrophil to Lymphocyte Ratio.
