# Supplementary figures and images for: Body mass index distinctly modulates the associations between Alistipes and CRP/IL-6 in metabolic and lupus inflammatory features
Source: PLoS One. 2025 Nov 25;20(11):e0335452. doi: 10.1371/journal.pone.0335452 (PMC12646403; doi:10.1371/journal.pone.0335452)

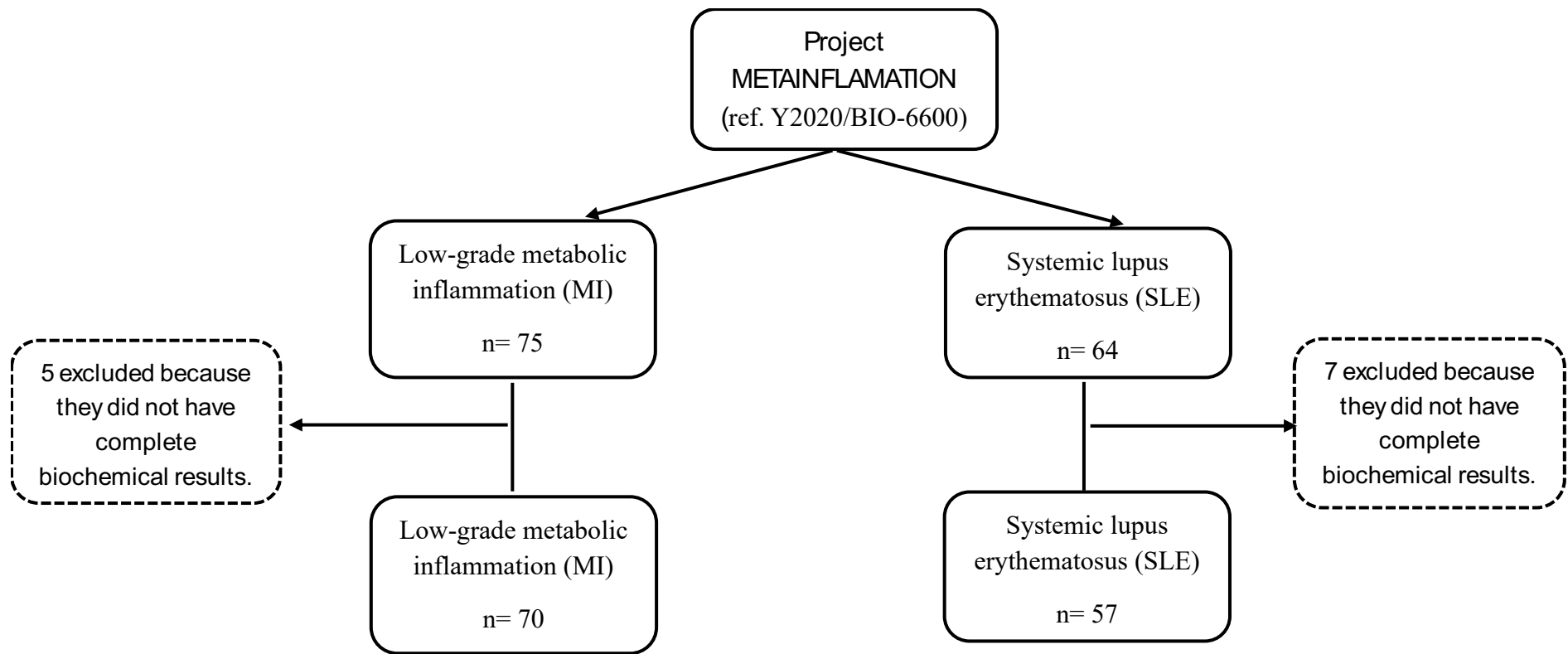

**Figure S1.** Flowchart of METAINFLAMATION project participants.

Supplement: S1 Fig — (PDF) [file pone.0335452.s004.pdf]
